# Molecular and Mechanistic Characterization of PddB, the First PLP-Independent 2,4-Diaminobutyric Acid Racemase Discovered in an Actinobacterial D-Amino Acid Homopolymer Biosynthesis

**DOI:** 10.3389/fmicb.2021.686023

**Published:** 2021-06-10

**Authors:** Kazuya Yamanaka, Ryo Ozaki, Yoshimitsu Hamano, Tadao Oikawa

**Affiliations:** ^1^Department of Life Science and Technology, Faculty of Chemistry, Materials, and Bioengineering, Kansai University, Suita, Japan; ^2^Graduate School of Science and Engineering, Kansai University, Suita, Japan; ^3^Department of Bioscience, Faculty of Biotechnology, Fukui Prefectural University, Yoshida-gun, Japan

**Keywords:** D-amino acid, PLP-independent, diaminopimelate epimerase, secondary metabolite, biosynthesis, homo poly-amino acid, 2, 4-diaminobutyric acid, amino acid racemase

## Abstract

We recently disclosed that the biosynthesis of antiviral γ-poly-D-2,4-diaminobutyric acid (poly-D-Dab) in *Streptoalloteichus hindustanus* involves an unprecedented cofactor independent stereoinversion of Dab catalyzed by PddB, which shows weak homology to diaminopimelate epimerase (DapF). Enzymological properties and mechanistic details of this enzyme, however, had remained to be elucidated. Here, through a series of biochemical characterizations, structural modeling, and site-directed mutageneses, we fully illustrate the first Dab-specific PLP-independent racemase PddB and further provide an insight into its evolution. The activity of the recombinant PddB was shown to be optimal around pH 8.5, and its other fundamental properties resembled those of typical PLP-independent racemases/epimerases. The enzyme catalyzed Dab specific stereoinversion with a calculated equilibrium constant of nearly unity, demonstrating that the reaction catalyzed by PddB is indeed racemization. Its activity was inhibited upon incubation with sulfhydryl reagents, and the site-directed substitution of two putative catalytic Cys residues led to the abolishment of the activity. These observations provided critical evidence that PddB employs the thiolate-thiol pair to catalyze interconversion of Dab isomers. Despite the low levels of sequence similarity, a phylogenetic analysis of PddB indicated its particular relevance to DapF among PLP-independent racemases/epimerases. Secondary structure prediction and 3D structural modeling of PddB revealed its remarkable conformational analogy to DapF, which in turn allowed us to predict amino acid residues potentially responsible for the discrimination of structural difference between diaminopimelate and its specific substrate, Dab. Further, PddB homologs which seemed to be narrowly distributed only in actinobacterial kingdom were constantly encoded adjacent to the putative poly-D-Dab synthetase gene. These observations strongly suggested that PddB could have evolved from the primary metabolic DapF in order to organize the biosynthesis pathway for the particular secondary metabolite, poly-D-Dab. The present study is on the first molecular characterization of PLP-independent Dab racemase and provides insights that could contribute to further discovery of unprecedented PLP-independent racemases.

## Introduction

Canonical D-amino acids, mirror-image isomers of proteinogenic L-amino acids, are widely distributed across the prokaryotic and eukaryotic kingdoms, and their biological roles are gradually being elucidated. For instance, D-serine and D-aspartate are known to be *N*-methyl-D-aspartate (NMDA) receptor co-agonists in mammals (Nishikawa, 2005), while D-alanine is a major osmolyte in bivalve and shrimp (Yoshikawa and Yokoyama, 2015). In prokaryotes, it is well known that D-alanine and D-glutamate are essential components of the cell wall peptidoglycan (Schouten et al., 2006) and are also seen in peptidic antibiotics produced as bacterial secondary metabolites. Recent studies further revealed the involvement of such canonical D-amino acids in the remodeling of peptidoglycan structure (Lam et al., 2009) and the dispersion of bacterial biofilms (Ampornaramveth et al., 2018). In general, free diffusible D-amino acids are generated by amino acid racemases catalyzing the interconversion of D- and L-amino acid enantiomers. These enzymes are classified into two groups based on their cofactor requirements: the pyridoxal 5′-phosphate (PLP)-dependent and -independent enzymes. The former require PLP and typically include alanine racemase (AlaR) (Inagaki et al., 1986) and arginine racemase (ArgR) (Yorifuji and Ogata, 1971). The later employ two catalytic Cys residues for the enantioinversion and include diaminopimerate epimerase (DapF) (Cirilli et al., 1998; Hor et al., 2010), glutamate racemase (GluR) (Yoshimura et al., 1993; Dodd et al., 2007), aspartate racemase (AspR) (Fujii et al., 2015; Washio et al., 2016), proline racemase (ProR) (Wu and Hurdle, 2014; Watanabe et al., 2015), and hydroxyproline epimerase (HypE) (Watanabe et al., 2017). While extensive studies carried out to date have elucidated the biological roles of such canonical D-amino acids and have identified numerous racemases responsible for their production, our knowledge of the non-canonical D-amino acids has largely remained limited owing to the scarcity of their biosynthetic and physiological precedents. Investigations into uncharacterized non-canonical amino acid racemases are thus expected to lead to an expansion of our knowledge of their structure-function relationships.

**FIGURE 1:**
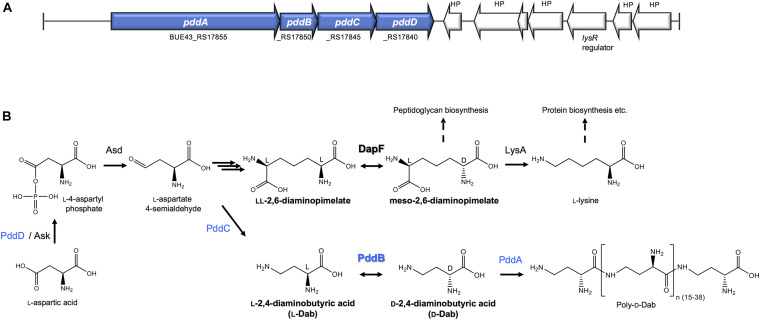
The biosynthesis of Poly-D-Dab. **(A)** Periphery of the genes involved in the Poly-D-Dab biosynthesis in *S. hindustanus* NBRC15115. The genes coding for enzymes responsible for the biosynthesis of Poly-D-Dab (PddA to PddD) are highlighted in blue. **(B)** Poly-D-Dab biosynthesis, which partially shares the primary metabolic lysine biosynthetic diaminopimelate pathway. Racemization of Dab catalyzed by secondary metabolic PddB and epimerization of diaminopimelate catalyzed by primary metabolic DapF are comparably shown. Ask, aspartate kinase; Asd, aspartate-semialdehyde dehydrogenase; LysA, meso-diaminopimelic acid decarboxylase.

A novel PLP-independent racemase that catalyzes the racemization of a non-canonical amino acid *O*-ureidoserine was discovered from the biosynthesis of D-cycloserine, an antibiotic produced as a secondary metabolite of *Streptomyces* strains (Dietrich et al., 2012). This demonstration was highly suggestive of the potential distribution of undiscovered non-canonical amino acid racemases in the biosynthetic pathways for unusual natural products, and thereby strongly motivated us to explore undiscovered racemases from the biosynthesis of the non-canonical D-amino acid homo-polymer, γ-poly-D-2,4-diaminobutyric acid (poly-D-Dab), in *Streptoalloteichus hindustanus.* As we recently disclosed, this unusual polymer with antiviral activity is biosynthesized by a distinctive membrane-bound single-module non-ribosomal peptide synthetase (NRPS)-like enzyme, poly-D-Dab synthetase (PddA) (Yamanaka et al., 2020). In this biosynthetic machinery, unlike in the canonical NRPSs, a free diffusible D-isomer of Dab directly undergoes adenylation activation and is subsequently condensed iteratively, yielding the polymer product with a D-configuration. Its biosynthetic precursor D-Dab is generated through the stereoinversion from its L-isomer by the catalytic activity of the protein product of the *pddB* gene translationally coupled with the *pddA* gene (Figures 1A,B). PddB exhibits weak primary structural similarity to PLP-independent epimerase DapF, which catalyzes the interconversion between L,L-diaminopimelic acid and meso-diaminopimelic acid in prokaryotic lysine biosynthesis (Figure 1B; Richaud et al., 1987; Koo and Blanchard, 1999; Hartmann et al., 2003). In the previous study, PddB was demonstrated to catalyze the interconversion of Dab isomers in a cofactor-independent manner (Yamanaka et al., 2020), whereas the enzyme remained wholly uncharacterized. A PLP-dependent ArgR from *Pseudomonas graveolens* has been shown to modestly catalyze the racemization of Dab because of its relaxed substrate selectivity (Yorifuji and Ogata, 1971). Since, to the best of our knowledge, PddB is the first enzyme capable of catalyzing the cofactor-independent stereoinversion of Dab, the enzymological properties and mechanistic details of this enzyme were of particular interest. In this study, we thus fully investigated the PddB through biochemical characterizations, *in silico* structural modelings, and site-directed mutageneses. Based on the findings from this series of investigations, we report here that the PddB is a PLP-independent racemase with strict specificity for Dab (DabR) and behaves similarly to the other racemases harboring a thiolate-thiol pair in the active site. Further, we provide insights into the evolution and structure-function relationships of DabRs.

## Materials and Methods

### Plasmids, Bacterial Strains, and Growth Conditions

pET-21b (+) (Merck/Novagen, Germany) was used for heterologous expression of the recombinant PddB and its homolog in combination with *Escherichia coli* Rosetta 2 (DE3) host cells. *E. coli* NEB10β (New England Biolabs, United States) was used for routine cloning. *S. hindustanus* DSM44523 (=NBRC15115) and *Actinokineospora enzanensis* DSM44649 (=NBRC16517) obtained from the NITE Biological Resource Center (NBRC) of Japan were maintained on ISP4 agar as described previously (Yamanaka et al., 2020). *E. coli* strains were routinely grown in Luria-Bertani (LB) medium. Carbenicillin (Carb, 150 μg/ml) and Chloramphenicol (Chl, 30 μg/ml) were used to select *E. coli* recombinant strains.

### Expression and Purification of Recombinant PddB and Its Homolog

The recombinant PddB (PID: WP_073484545.1) was expressed and purified as reported previously (Yamanaka et al., 2020). The recombinant PddB homolog from *A. enzanensis* (PID: WP_018680613.1) was similarly prepared. Briefly, the gene C503_RS0102450 coding for the PddB homolog was PCR-amplified from the genome of *A. enzanensis* NBRC16517 with the primers 018680613-ndeF (5′-AGATATACATATGGACATCGACCCGCGCGCGCTCGAACT C-3′) and -xhoR (5′-GGTGGTGCTCGAGGGGTGTCGCGCTC ACCGGGGCGTCCATGGTG-3′). The resulting PCR fragment was double digested with *Nde*I and *Xho*I and was subsequently ligated with similarly digested pET21b(+) vector, yielding the PddB homolog expression vector. After sequence verifications, this vector was introduced into *E. coli* Rosetta 2 (DE3) by electroporation, and the resulting cells were streaked out onto LB agar plates supplemented with Carb and Chl. A transformant was inoculated into 100 ml LB medium supplemented with Carb, Chl, and 0.4% (w/v) glucose and grown at 37°C with shaking until the OD_600_ reached around 0.6. After dropping the cultivation temperature down to 18°C, expression of the PddB homolog was induced by 0.2 mM isopropyl-β-D-thiogalactopyranoside (IPTG) for 16 h. Cells harvested were resuspended in buffer A consisting of 50 mM potassium phosphate buffer (pH 7.5), 300 mM NaCl, 10% (w/v) glycerol, 0.05% (w/v) triton X-100, and 10 mM imidazole, and were sonicated and centrifuged to yield the cell free extract. The supernatant was applied onto a Ni-NTA agarose column (Qiagen, United States) pre-equilibrated with buffer A. Fractions eluted with buffer A containing 50 to 200 mM imidazole were pooled. The purity-verified recombinant enzyme on sodium dodecyl sulfate-polyacrylamide gel electrophoresis (SDS-PAGE) was dialyzed against 50 mM potassium phosphate buffer (pH 7.5) containing 10% (w/v) glycerol. The protein concentration was determined using a Bio-Rad Protein assay kit (Bio-Rad) with BSA as the standard. The purified enzyme was stored at −80°C until use.

### Construction of PddB Mutants by PCR-Mediated Site-Directed Mutagenesis

The single point mutations were introduced into the expression plasmid pET21b-*pddB*, which we previously constructed (Yamanaka et al., 2020), by the PCR-mediated QuickChange mutagenesis strategy (Zheng et al., 2004). The plasmid carrying the C101S mutation on the *pddB* gene was generated by PCR using a set of overlapping oligo nucleotide primers pddB-C101S-F (5′-ACGCCCAGATG**A**GCGGCAACGCGCTGCGCTGCCTG-3′) and -C101S-R (5′-GCGCGTTGCCGC**T**CATCTGGGCGTGCGT TCCGTC-3′). Similarly, the mutation corresponding to C233S was introduced with the primers pddB-C233S-F (5′-AGACCAAGGCC**A**GTGGGACCGGCGTCGCCGCGAC-3′) and -C233S-R (5′-CGCCGGTCCCAC**T**GGCCTTGGTCTCGTC CTCCAC-3′). Overlapping sequences for self-annealing are underlined and the substituted nucleotides are in bold. After sequence verifications, the mutated plasmids were individually introduced into *E. coli* Rosetta (DE3), resulting in the expression of two single point mutants of PddB under the established cultivation/expression conditions. The mutants were purified using Ni-NTA agarose resin as described above.

### Physicochemical Characterizations of PddB

The molecular weight of PddB was determined by size-exclusion chromatography using an ÄKTA purifier system equipped with a Superdex 200 10/300 GL column (GE Healthcare, Japan). The mobile phase was an isocratic flow of 50 mM potassium phosphate buffer (pH 7.5) containing 0.15 M KCl at a rate of 0.75 ml/min, and eluents were spectrophotometrically monitored at 210 nm. The calibration curve used for the molecular weight determination was established with aprotinin (6.5 kDa), ribonuclease (13.7 kDa), carbonic anhydrase (29 kDa), ovalbumin (44 kDa), conalbumin (75 kDa), aldolase (158 kDa), and ferritin (440 kDa).

The UV-vis absorption spectrum of PddB dissolved in 50 mM potassium phosphate buffer (pH 7.5) containing 0.15 M KCl was analyzed using V-730BIO spectrophotometer (Jasco corporation, Japan).

### Enzyme Assays

All the DabR assays were performed with 0.2 ml reaction mixtures. In the standard assay, the reaction mixture was simply comprised of 100 mM TAPS-NaOH (pH 8.5) and 20 mM enantiopure Dab. Reactions were initiated by addition of 5 μg purified enzyme, and after incubation at 30°C, 20 μl of 50% (w/v) trichloroacetic acid was added to terminate the reaction. The supernatant was neutralized with 100 μl of 0.5 M NaOH and subjected to the quantitative enantio-resolution employing Marfey’s method (Radkov and Moe, 2013) with slight modifications as follows. To a portion (50 μl) of the product, 100 μl of 1% (w/v) 1-fluoro-2,4-dinitrophenyl-5-L-leucine amide (L-FDLA) in acetone and 20 μl 1 M NaHCO_3_ were added. To yield the di-FDLA-labeled product, the mixture was incubated at 37°C for 24 h under dark condition. After quenching the derivatization by addition of 20 μl of 1 M HCl, the resulting solution was diluted with 810 μl of methanol and placed into an amber glass vial prior to the quantitative enantioresolution in the Shimadzu LC-MS-IT-TOF instrument equipped with the Prominence HPLC system. The LC separation was performed using a C18 reverse-phase column (SunShell C18, 2.6 μm, 2.1 × 50 mm, ChromaNik Technologies Inc., Japan) at 40°C with a binary solvent system consisting of 0.03% trifluoroacetic acid plus 0.07% formic acid in H_2_O (A) and in acetonitrile (B). FDLA-derivatized enantiomers separated with a linear gradient of 45–65% B over 15 min at a flow rate of 0.3 ml/min were monitored at 340 nm and were eluted into the electrospray ionization (ESI) interface in positive mode. LC-MS Solution software (Shimadzu, Japan) was used for system control and data analysis. Authentic standards of D- and L-Dab were used to establish standard curves for quantification. The racemase activity was determined from the initial velocity of the stereoinversion reaction, and one enzyme unit (U) was defined as the amount of the enzyme that catalyzes the conversion of one micromole of substrate per minute. All the assays were performed in triplicate.

The substrate specificity of PddB was examined using 20 mM concentration of L-arginine, L-lysine, L-ornithine, L-2,3-diaminopropionic acid, LL-2,6-diaminopimelate, and their corresponding enantiomers under the standard assay conditions. The effect of pH on PddB activity was evaluated by measuring the racemization from L-Dab under various pH conditions at 30°C. The reaction mixtures for this evaluation contained 100 mM HEPES-NaOH (pH 7.0–8.0), TAPS-NaOH (pH 8.0–9.0), or CHES-NaOH (pH 8.5–9.5). To evaluate the inhibitory effect, PddB was individually incubated with chelating agents, metal ions, sulfhydryl reagents, or other compounds in 0.2 ml of 100 mM TAPS buffer (pH 8.5) at 30°C. After incubation for 20 min, residual activity was evaluated by measuring the racemization from L-Dab under the standard condition. For determination of kinetic parameters, measurements obtained under the standard condition with various concentration of each Dab isomer were processed using a Lineweaver-Burk plot (Lineweaver and Burk, 1934).

### Molecular Modeling

The SWISS-MODEL server^1^ (Kiefer et al., 2009) was used for secondary structure prediction and for the generation of a 3D structure model of PddB with the crystal structure of DapF from *Haemophilus influenzae* (Hi-DapF, PDB ID: 2GKE) (Pillai et al., 2006) as template.

## Results

### Expression, Purification, and Physicochemical Characterization of PddB

**FIGURE 2:**
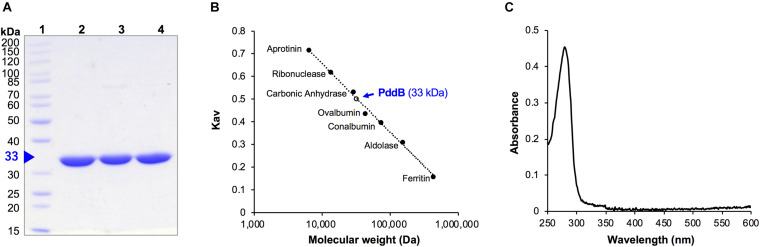
Purification and physicochemical characterization of PddB. **(A)** Purity of wild-type PddB and its mutants evaluated in this study. The purified proteins were analyzed on SDS-PAGE and stained with Coomassie Brilliant Blue R-250. Lane 1, molecular weight marker; lane 2, wild-type PddB; lane 3, C101A mutant; lane 4, C233S mutant. **(B)** Elution behavior of PddB on size-exclusion chromatography. **(C)** Absorption spectrum of PddB.

As we previously reported, the C-terminally His_6_-tag fused recombinant PddB (PID: WP_073484545.1) was expressed in *E. coli* mostly as a soluble protein and was confirmed to catalyze the stereoinversion of Dab (Yamanaka et al., 2020). However, it was uncertain whether the stereoinversion was a racemization, in the strictest sense of the term. In addition, the physicochemical and enzymological properties of the enzyme have yet to be elucidated. To this end, we first expressed and purified recombinant PddB according to the reported procedures (Figure 2A). The purified PddB was subsequently subjected to physicochemical analyses. Both in SDS-PAGE and size-exclusion chromatography, its molecular weight was determined to be 33 kDa (Figures 2A,B), demonstrating that PddB behaves as a monomeric enzyme. In UV-spectrometric analysis of the purified PddB, as shown in Figure 2C, the characteristic absorption of the PLP bound enzyme (around 420 nm) was undetectable. This observation suggested that the enzyme is a PLP-independent racemase that employs two catalytic Cys residues.

### Biochemical Characterization of PddB

For racemase assays, in this study, we applied the advanced Marfey’s method using L-FDLA as a pre-column derivatization agent as reported previously (Radkov and Moe, 2013). In our preliminary study using various diamino acid authentic standards including Dab, LC-MS analysis revealed that a commonly employed derivatization condition (40°C for 1 h) produces both mono- and di-FDLA labeled derivatives and thus makes the measurements unsettled, while prolonged derivatization (37°C for 3–24 h) led to the complete conversion to di-FDLA labeled derivatives, which in turn brought about a precise quantitative enantio-determination. We thus employed this optimized derivatization condition for quantitative analysis of the stereoinversion reaction catalyzed by PddB.

**FIGURE 3:**
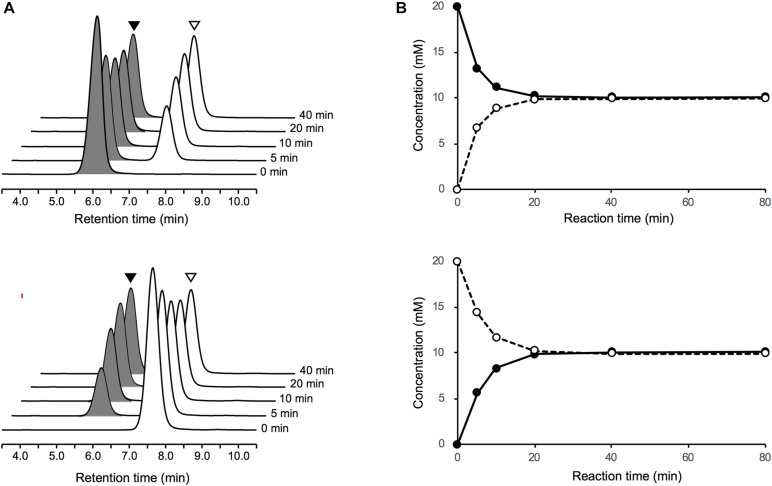
LC-MS analysis of Dab racemase reactions catalyzed by PddB. **(A)** Stereoinversions from 20 mM of L-Dab (black arrowheads) and from D-Dab (white arrowheads) monitored at 340 nm are shown the in upper and lower panels, respectively. All peaks presented exhibited 707.3 m/z as (M + H)^+^, which corresponded to Dab doubly labeled with FDLA, and no singly labeled Dab was detected. **(B)** Transition of the stereoinversion reactions from 20 mM of L-Dab (closed circle with a solid line) and from D-Dab (open circle with a dotted line) calculated based on the LC chromatograms are shown in the upper and lower panels, respectively.

Upon incubation of the purified PddB with each of the Dab enantiomer in the absence of PLP, the stereoinversions rapidly reached an equilibrium state (Figure 3), and the activity of the purified PddB was affected by administration of neither PLP nor hydroxylamine (Table 1), demonstrating its cofactor independence. We next set out to investigate the enzymological properties of PddB. Similar to the canonical PLP-independent racemases, the enzyme was revealed to prefer weak alkaline pH conditions for its catalysis. Since PddB exhibited maximum activity at around pH 8.5 in the presence of 20 mM Dab (Supplementary Figure 1A), these parameters were used for the following enzymological characterizations unless otherwise noted. The stereoinversion of Dab gradually accelerated with increasing reaction temperature and reached maximum at 50°C (Supplementary Figure 1B).

**TABLE 1:** Inhibitory effect of various compounds on PddB activity.

Compounds	Remaining activity (%)
None	100
PLP (1 mM)	112 ± 2.1
Hydroxylamine (10 μM)	82 ± 0.4
EDTA (1 mM)	111 ± 0.4
MgCl_2_ (1 mM)	103 ± 4.2
MnCl_2_ (1 mM)	40 ± 0.1
HgCl_2_ (10 μM)	0
IAA (20 μM)	45 ± 6.4
IAM (20 μM)	38 ± 0.9
PCMB (10 μM)	0

*The activity (L → D) was determined after incubation with each agent for 20 min at 30°C.*

To determine the kinetic parameters of PddB, the initial velocities of the PddB reaction under various concentration of Dab isomers were assayed. PddB was shown to exhibit typical Michaelis–Menten kinetics (Supplementary Figure 2). As shown in Table 2, in the L to D stereoinversion, the enzyme displayed significantly higher *V*_max_, *K*_*m*_, and *k*_cat_ values than those in the reverse reaction. These characteristic kinetic profiles of PddB implied that the velocity of the interconversion reaction would be biased toward formation of the D-Dab precursor for Poly-D-Dab biosynthesis under the sufficient levels of intracellular L-Dab furnished by the associated biosynthetic enzymes, pathway-specific aspartate kinase PddD and diaminobutyrate-2-oxoglutarate transaminase PddC (Yamanaka et al., 2020). Meanwhile, specificity constants (*k*_cat_/*K*_m_) for each reaction direction were shown to be nearly equal. In addition, based on the conclusive observation that the equilibrium constant *K*_*eq*_ of the PddB reaction calculated from the *V*_max_/*K*_m_ values between both reaction directions was near unity (1.07), the stereoinversion reaction catalyzed by PddB was demonstrated to indeed be the racemization (Briggs and Haldane, 1925).

**TABLE 2:** Kinetic parameters of PddB.

Parameters	Reaction direction	
	L → D	D → L
*V*_*max*_ (U mg^–1^)	210 ± 21	136 ± 8
*K*_*m*_ (mM)	10.1 ± 1.9	6.1 ± 1.0
*k*_*cat*_ (s^–1^)	116 ± 12	75 ± 5
*k*_*cat*__/_*K*_*m*_ (s^–1^mM^–1^)	11.4	12.2
*V*_*max*_/*K*_*m*_ (U mg^–1^mM^–1^)	20.8	22.2

As we reported previously, PddB accepted neither LL-2,6-diaminopimelate nor meso-2,6-diaminopimelate as a substrate. In this study, various amino acids including diamino acid analogs of Dab were tested to fully unveil its substrate specificity. These assays demonstrated that PddB exhibits strict selectivity—namely, L-arginine, L-lysine, L-ornithine, L-2,3-diaminopropionic acid, and their corresponding enantiomers were all inert as substrates (data not shown).

As summarized in Table 3, the fundamental properties of PddB elucidated herein, such as its monomeric structure, basophilic property, and strict substrate recognition, were in good agreement with those of typical PLP-independent racemases, including the well-characterized DapF from *Mycobacterium tuberculosis* (Mt-DapF) (Usha et al., 2008), which employs two catalytic Cys residues for enantioinversion (Cardinale and Abeles, 1968; Richaud et al., 1987; Yoshimura et al., 1993; Koo and Blanchard, 1999; Hartmann et al., 2003; Fujii et al., 2015).

**TABLE 3:** Comparison of enzymological properties between DabRs and Mt-DapF.

	PddB	PddB homolog (*A. enzanensis*)	Mt-DapF^c^
Molecular weight^a^	33 kDa	32 kDa	30 kDa
Quaternary structure^b^	Monomeric	Monomeric	Monomeric
Cofactor requirement	No	No	No
Substrate specificity	Dab specific	Dab specific	Diaminopimelic acid specific
Optimum pH	8.5	8.5	7.5
Optimum temperature	50°C	N.D.	30°C

*^*a*^Estimated by SDS-PAGE.*

*^*b*^Determined by size-exclusion chromatography.*

*^*c*^Properties of Mt-DapF are from the published literature.*

### Effect of Sulfhydryl Reagents on PddB Activity

To determine whether PddB operates *via* the thiolate-thiol pair within the active site similar to the canonical PLP-independent racemases, we assessed the impact of sulfhydryl reagents on PddB activity. As shown in Table 1, the activity of PddB was almost completely abolished upon incubation with sulfhydryl reagents such as HgCl_2_ and 4-chloromercuribenzoic acid (PCMB) at concentrations of 10 μM, and was dramatically decreased with 20 μM of iodoacetic acid (IAA) or iodoacetamide (IAM). These observations thus strongly suggested that PddB most likely relies on Cys residues for the catalysis like the other PLP-independent racemases/epimerases (Fischer et al., 2019). In contrast, the activity of PddB was not affected by the incubation with metal ions or ethylenediaminetetraacetic acid (EDTA), indicating its metal-independence.

### In silico Structural Prediction of PddB

**FIGURE 4:**
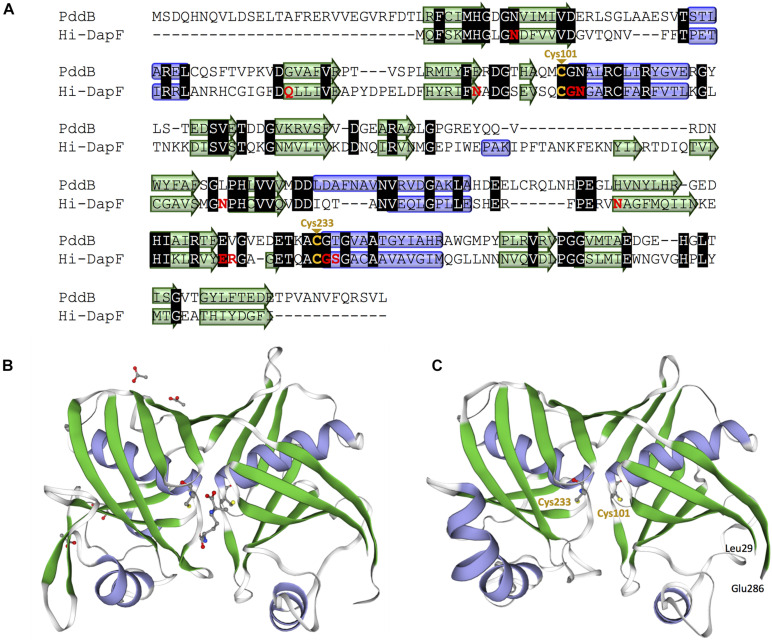
Amino acid sequence and structural models of PddB. **(A)** Amino acid sequence alignment of PddB and DapF from *Haemophilus influenzae* (Hi-DapF) with their secondary structural comparison. Helices and sheets predicted by the Dictionary of Secondary Structure of Proteins (DSSP) algorithm are marked as blue tubes and green arrows, respectively. In the Hi-DapF sequence, amino acid residues which have been reported to interact with the substrate *via* hydrogen bonds or a salt bridge are highlighted in red. **(B)** 3D structure of Hi-DapF in complex with an irreversible inhibitor LL-AziDAP (PDB ID: 2GKE). **(C)** 3D structural model of PddB. The model was built in the SWISS-MODEL server using the structure of Hi-DapF as a template.

In the genome of *S. hindustanus*, in addition to the *pddB* gene, the gene tagged BUE43_RS11395 has also been annotated as *dapF* based on the sequence similarity. While PddB showed quite weak sequence homology to Mt-DapF (27% identical), the other protein was revealed to exhibit higher homology (55% identical), and thus this protein was presumed to function as the primary metabolic DapF in *S. hindustanus*. Despite such disparity in their primary structures, a PROSITE motif search (Hulo et al., 2006) for PddB identified a nearly conserved DapF active site signature N-x-(DN)-(GS)-(SENGFT)-x(4)-C-(GI)-N-(GA)-x-R (PROSITE PDOC01029) including the putative catalytic Cys residue. Sequence comparison with the well-studied DapF from *H. influenzae* (Hi-DapF, PID: WP_005655521) further identified the other putative catalytic Cys conserved within the C-terminal side of PddB (Figure 4A). The secondary structure of PddB predicted by using the Dictionary of Secondary Structure of Proteins (DSSP) algorithm was also in good agreement with that of Hi-DapF. Furthermore, a 3D structural model of PddB built on the SWISS-MODEL server using the structure of Hi-DapF in complex with an aziridino analogs of diaminopimelate LL-AziDAP (PDB ID: 2GKE) (Pillai et al., 2006) as template suggested that the overall structure of PddB was quite similar to that of Hi-DapF (Figures 4B,C). In particular, it appeared that they share a distinctive pseudo-superimposable di-domain structure having adjacently arranged catalytic Cys residues at the domain interface. All these structural predictions suggested that Cys_101_ and Cys_233_ in PddB could form a catalytic diad.

### Identification of Catalytic Residues in PddB by Site-Directed Mutagenesis

**FIGURE 5:**
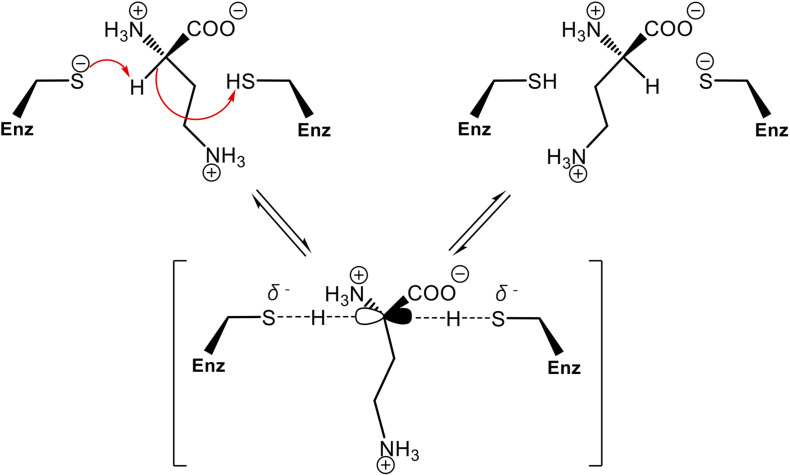
Proposed reaction mechanism of PddB that employs the thiolate-thiol pair in the active site. The two catalytic Cys residues interconvert L-Dab and D-Dab through a transient planar carbanion intermediate as indicated within parentheses. This intermediate can be derived from either isomer. Fundamentally, the base (thiolate) deprotonates the substrate C^α^, which can then be protonated *via* the acid (thiol) of the other Cys residue. The arrows represent electronic rearrangements relating to the racemization.

In order to assess whether the Cys residues are involved in PddB catalysis, two single-point mutants (C101S and C233S) were individually generated. Like the wild-type PddB, the mutant enzymes were mostly expressed as soluble proteins, and the purification in an identical fashion resulted in a comparable yield (Figure 2A). These observations indicated that the mutant enzymes retained the normal conformation, and thus we validated their catalytic activities. The enzyme assays revealed that the substitution of either Cys residue almost completely abolished the activity for both reaction directions (data not shown). In the prolonged reactions, however, a hardly quantifiable but faintly remaining activity (less than 0.1% of the wild-type) was observed as reported in Cys → Ser substituted Mt-DapF and other PLP-independent racemases (Usha et al., 2008; Glavas and Tanner, 1999). Considering the differences in p*K*_*a*_ values between a free thiol (p*K*_*a*_ ≈ 10) and a hydroxyl (p*K*_*a*_ ≈ 16), the dramatically decreased activities observed in the mutants well supported the proposed reaction mechanism of PddB shown in Figure 5, which employs the thiolate-thiol pair for racemization of Dab.

## Discussion

Our recent investigation into the biosynthesis of the antiviral cationic homo poly-amino acid poly-D-Dab revealed that the DapF homolog PddB encoded in its biosynthetic gene cluster catalyzes stereoinversion of Dab in a cofactor-independent manner. The fact that cofactor-independent racemization of Dab had yet to be reported strongly motivated us to fully characterize this enzyme. In this study, through a series of biochemical characterizations, structural modeling, and site-directed mutageneses, we successfully demonstrated that PddB is indeed an unprecedented DabR, which employs a two-base mechanism like the other PLP-independent racemases/epimerases (Richaud et al., 1987; Yoshimura et al., 1993; Koo and Blanchard, 1999; Hartmann et al., 2003; Fujii et al., 2015).

**FIGURE 6:**
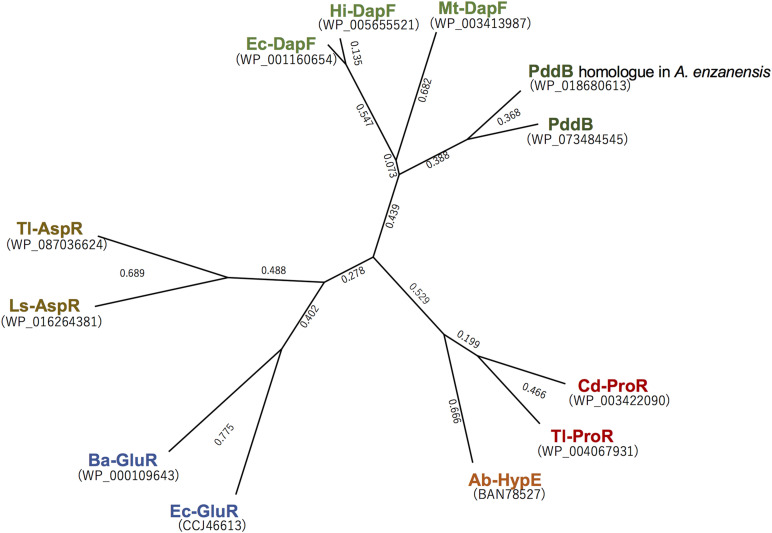
Phylogenetic tree analysis of the selected PLP-independent racemases/epimerases. The tree was generated in Genetyx software with the Unweighted Pair Group Method using the arithmetic Average (UPGMA) method. Ba-GluR, glutamate racemase from *Bacillus anthracis*; Ec-GluR, glutamate racemase from *Escherichia coli*; Ls-AspR, aspartate racemase from *Lactobacillus sakei*; Tl-AspR, aspartate racemase from *Thermococcus litoralis*; Hi-DapF, DapF from *Haemophilus influenzae*; Ec-DapF, DapF from *E. coli*; Cd-ProR, proline racemase from *Clostridioides difficile*; Tl-ProR, proline racemase from *T. litoralis*; Ab-HypE, L-hydroxyproline 2-epimerase from *Azospirillum brasilense*.

Although a PLP-dependent ArgR from *P. graveolens* has been reported to modestly catalyze the racemization of Dab because of its broad substrate specificity (Yorifuji and Ogata, 1971), Dab may not be a physiological substrate of this enzyme *in vivo*. In contrast, PddB characterized in this study selectively catalyzed the interconversion of Dab isomers. This strict selectivity would contribute to the organization of a particular biosynthetic pathway for the secondary metabolite, poly-D-Dab. In fact, a BLAST search using the amino acid sequence of PddB as a query was able to discriminatively identify three probable DabRs among the DapF homologs based on their homology; WP_158890712.1 in *Amycolatopsis anabasis* EGI 650086, WP_018680613.1 in *A. enzanensis* DSM44649, and WP_104476183.1 in *Actinokineospora auranticolor* YU 961-1 exhibited 56.6, 55.0, and 53.3% identities to the PddB, respectively. Furthermore, each gene coding for these three PddB homologs was, similar to the *pddB* gene, translationally coupled with the gene coding for poly-D-Dab synthetase (PddA) homolog (Supplementary Figure 3), suggesting that they could also function as DabRs. Indeed, among these, the recombinant protein of the PddB homolog from *A. enzanensis* was found to display the expected DabR activity with physiological and biochemical properties consistent with those of PddB (Supplementary Figure 4). A primary structure-based phylogenetic analysis among PLP-independent racemases/epimerases indicated a clear evolutionary relationship between the DabR and DapF enzymes (Figure 6). It has been postulated that some secondary metabolic pathways, for instance, the biosynthesis of lignin in tracheophytes, could have recruited primary metabolic enzymes to emerge and develop the pathway (Weng and Chapple, 2010). All these observations thus strongly suggest the possibility that DabRs could have been recruited and specially evolved from primary metabolic DapF in order to organize the biosynthesis pathway for the particular secondary metabolite poly-D-Dab.

Multiple sequence alignment revealed a distinctive peptidic extension (27–28 residues) at the N-terminal of DabRs (Supplementary Figure 5). The 3D modeling of PddB predicted that this N-terminal tail is more likely located on the surface of its pseudo-superimposable di-domain structure. Furthermore, as shown in Supplementary Figure 6, the secondary structure of the N-terminal domain without the tail (position 29–162) and the C-terminal domain (position 163–295) exhibited significant similarity with each other, indicating that the catalytic di-domain structure could be formed exclusively by those core regions. Considering that DabRs selectively recognize and stereoinvert the diamino acid substrate Dab, which is relatively smaller than that for DapF, the N-terminal tail might serve as a lid that wraps and narrows the active site within the di-domain cleft. Otherwise, the tail may contain amino acid residues that serve as some sort of docking anchor to specifically form an enzyme complex with its counterpart poly-D-Dab synthetase PddA (Rajamani et al., 2004). It has been suggested that PLP-independent racemases/epimerases have evolved through progressive adaptations of their symmetric di-domain structure (Liu et al., 2002). Thus, we suppose that DabRs have probably acquired such specialized structural features during the evolutionary process from ancestral DapF enzymes in order to adapt them to the pathway-specific racemase.

**FIGURE 7:**
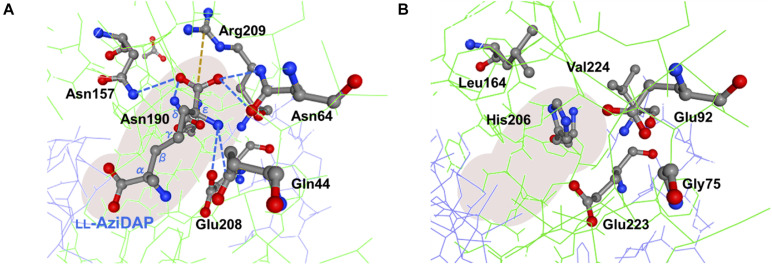
Predictive active site model for PddB. **(A)** Schematic representation of interactions between the non-reacting stereocenter of LL-AziDap and the six amino acid residues within the active-site cavity of Hi-DapF (PDB ID: 2GKE). Hydrogen bonds and a salt bridge are shown in blue dotted lines and brown dotted line, respectively. **(B)** Active site model for PddB generated in the SWISS-MODEL server using the structure of Hi-DapF as a template. Configuration of amino acid residues corresponding to the six active site residues in Hi-DapF are schematically shown.

As noted, despite PddB’s weak sequence similarity to DapF enzymes, a nearly conserved DapF active site signature and a small number of clearly conserved residues around its active site can be seen (Supplementary Figure 5). In Hi-Dap, four amino acid residues Gly74, Asn75, Gly218, and Ser219 lining the active-site cavity form hydrogen bonds with the carboxyl group at the stereocenter (reactive α-carbon) of diaminopimelate (Dap) (Pillai et al., 2006). Similarly, Asn11 interacts with the reactive α-amino group of Dap *via* hydrogen bonding. The structural model of PddB generated using the X-ray crystal structure of Hi-DapF as a template revealed the conservation of all the five residues (Supplementary Figure 5). As shown in Figure 7, Glu208 which interacts with the distal (non-reactive) ε-amino group of the substrate Dap was also shown to be conserved in PddB with the slightly dislocated orientation of the side chain, whereas the other hydrogen bonding residue Gln44 appeared to be substituted to Gly. Notably, although in Hi-DapF, Asn64, Asn157, and Asn190 form hydrogen bonds with the distal ε-carboxyl group of Dap, the structural model of the PddB indicated that the corresponding residues were all substituted with non-equivalent residues. Further, Arg209 in Hi-DapF that interacts with the carboxyl group at the non-reactive distal stereocenter of Dap *via* a salt bridge appeared to be substituted by hydrophobic Val in PddB. Since, all these substitutions are universally conserved in the other DabRs (Supplementary Figure 5), these distinctive residues in the active-site cavity of PddB could be responsible for the intolerance to the distal carboxyl group and the distended methylene chain of diamino acid substrate. Mutational analyses and crystallographic studies would provide insight into the structure-specificity relationship in PddB, the first PLP-independent racemase characterized herein. Eventually, knowledge of the mechanistic details of DabRs acquired through such investigations will facilitate the further discovery of unprecedented PLP-independent racemases specific for rare non-canonical amino acids and certain diamino acids such as lysine and ornithine, and even *de novo* creation of novel racemases by evolutionary engineering approaches. Our current study should thus provide valuable insights into PLP-independent racemases.

## Data Availability Statement

The original contributions presented in the study are included in the article/Supplementary Material, further inquiries can be directed to the corresponding author/s.

## Author Contributions

KY designed the experiments, analyzed the experimental data, and organized the manuscript. TO and YH assisted in the experimental design and performed the data analysis. RO conducted the experiments. Each author substantially contributed to the work reported herein.

## Conflict of Interest

The authors declare that the research was conducted in the absence of any commercial or financial relationships that could be construed as a potential conflict of interest.

## Footnotes

1 http://swissmodel.expasy.org/
