# Supplementary material for: Molecular and Mechanistic Characterization of PddB, the First PLP-Independent 2,4-Diaminobutyric Acid Racemase Discovered in an Actinobacterial D-Amino Acid Homopolymer Biosynthesis
Source: Front Microbiol. 2021 Jun 10;12:686023. doi: 10.3389/fmicb.2021.686023 (PMC8225329; doi:10.3389/fmicb.2021.686023)
Supplement: Supplementary file 1 [file Data_Sheet_1.PDF]

Supplementary Material

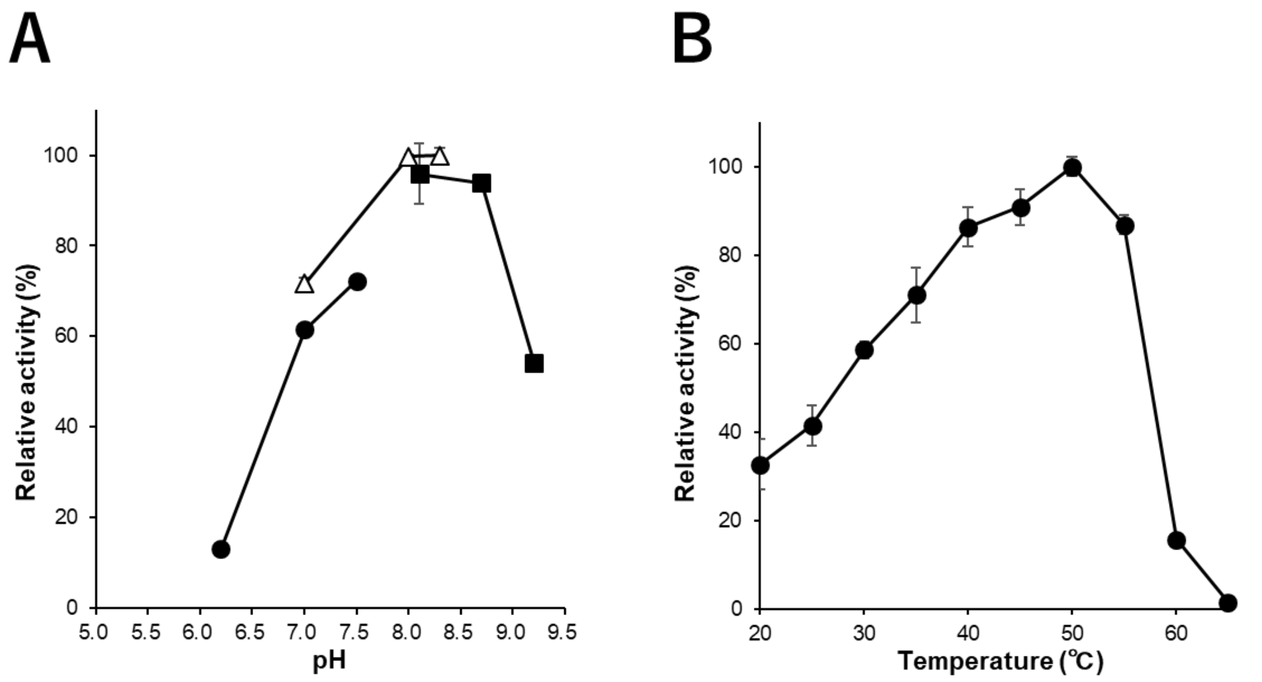

Supplementary Figure S1. pH and thermal profiles of PddB. (**A**) Effect of pH on DabR activity. The activities were evaluated in 100 mM HEPES-NaOH (closed circles), 100 mM TAPS-NaOH (open triangles), and 100 mM CHES-NaOH (closed squares), respectively. (**B**) Effect of temperature on DabR activity. The activities in TAPS-NaOH (pH 8.5) were assessed from 20℃ to 65℃.

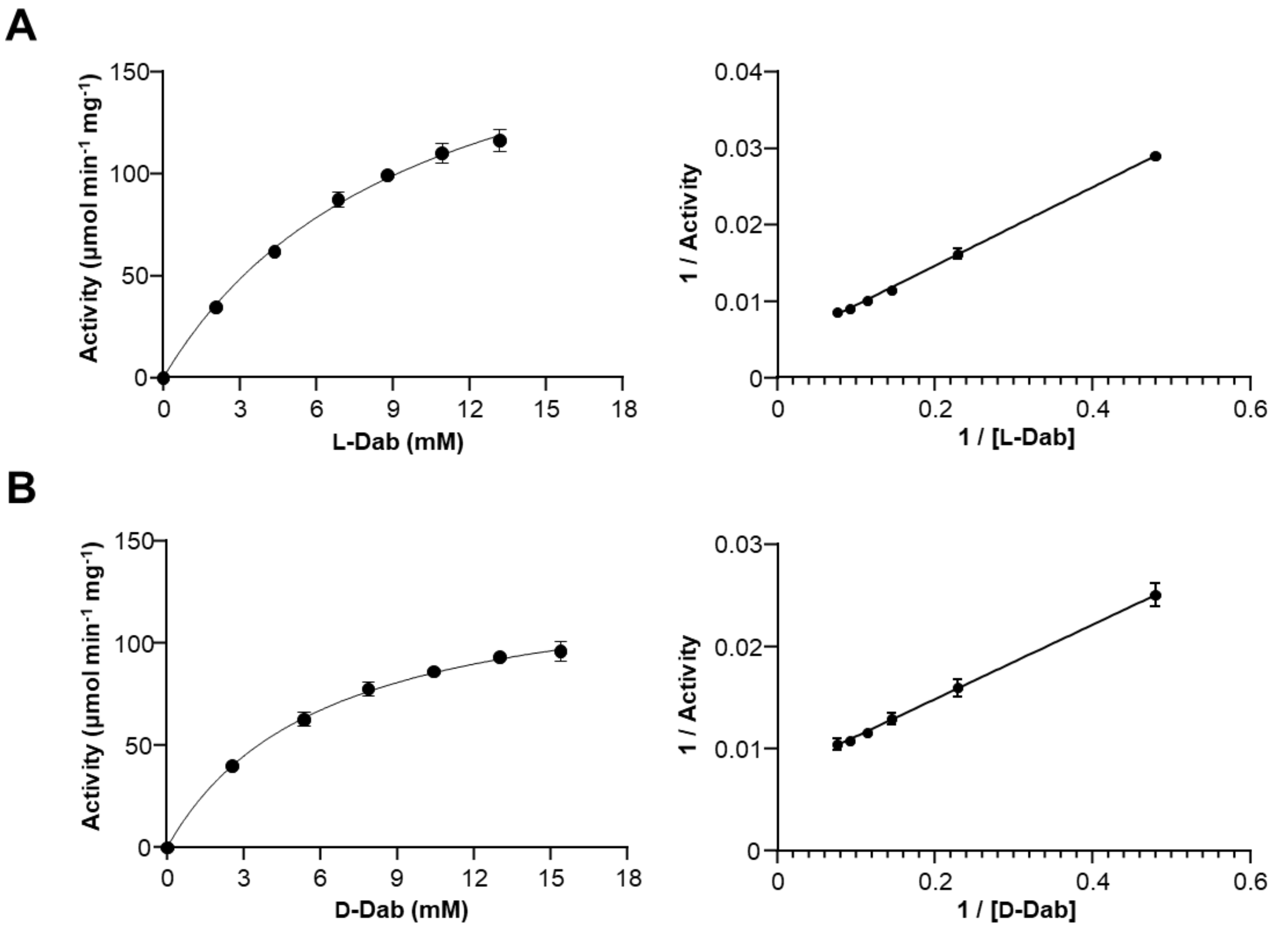

Supplementary Figure S2. Kinetic analysis of PddB. (**A**) Kinetic profile for L-Dab. (**B**) Kinetic profile for D-Dab. The initial velocity against the substrate concentration plots with nonlinear fitting into the Michaelis−Menten equation and the double-reciprocal plots are shown in the left and right panels, respectively.

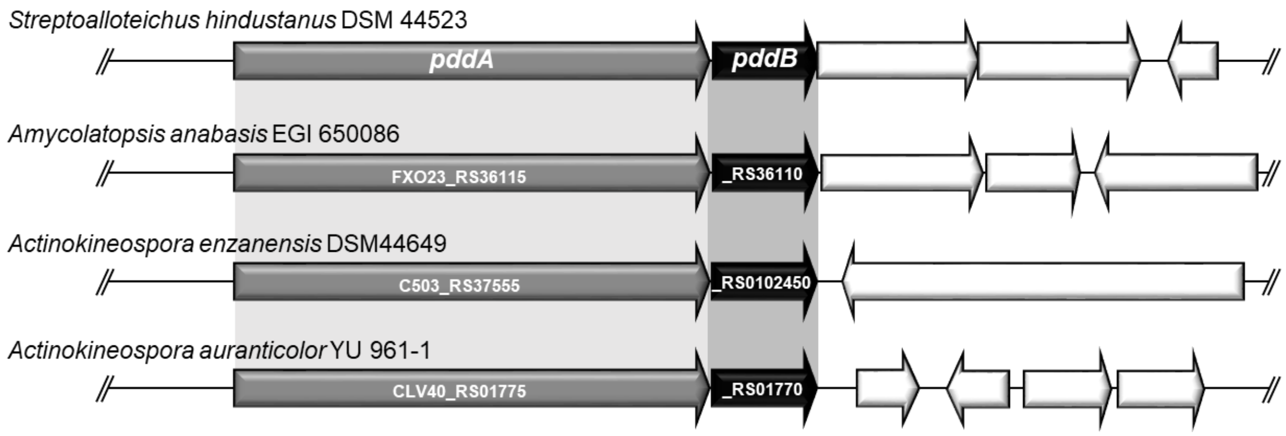

Supplementary Figure S3. The poly-D-Dab biosynthetic gene organization (*pddA*-*pddB*) in *S. hindastanus* and periphery of the PddB homolog coding genes in the other Actinobacterial strains.

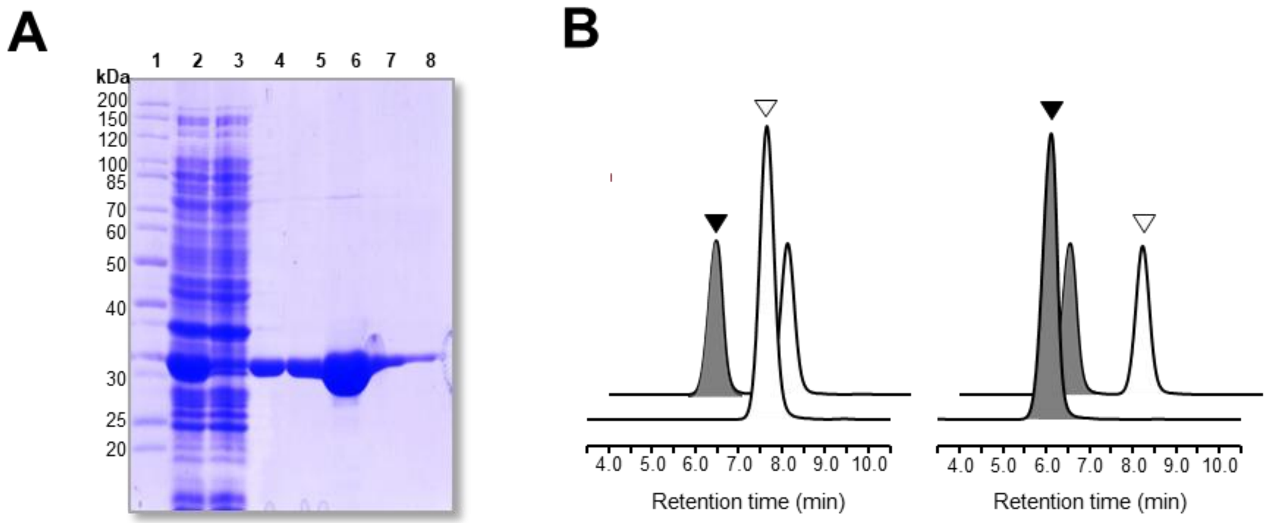

Supplementary Figure S4. Expression, purification, and analysis of the PddB homolog from *A. enzanensis*. (**A**) SDS-PAGE analysis of the recombinant PddB homolog expressed in *E. coli* Rosetta (DE3). Lane 1, molecular weight marker; lane 2, cell-free extract; lane 3, flow-through of Ni-affinity chromatography; lanes 4 to 8, Ni-affinity chromatography-purified fractions. (**B**) LC-MS analysis of Dab racemase reactions. The stereoinversion from L- and D-isomers monitored at 340 nm are shown for comparison. In both reactions, the stereoinversion reached an equilibrium state rapidly. All peaks exhibited 707.3 m/z as $[M+H]^+$, which corresponded to Dab doubly labeled with FDLA. White and black arrowheads indicate di-FDLA labeled D-Dab and L-Dab, respectively.

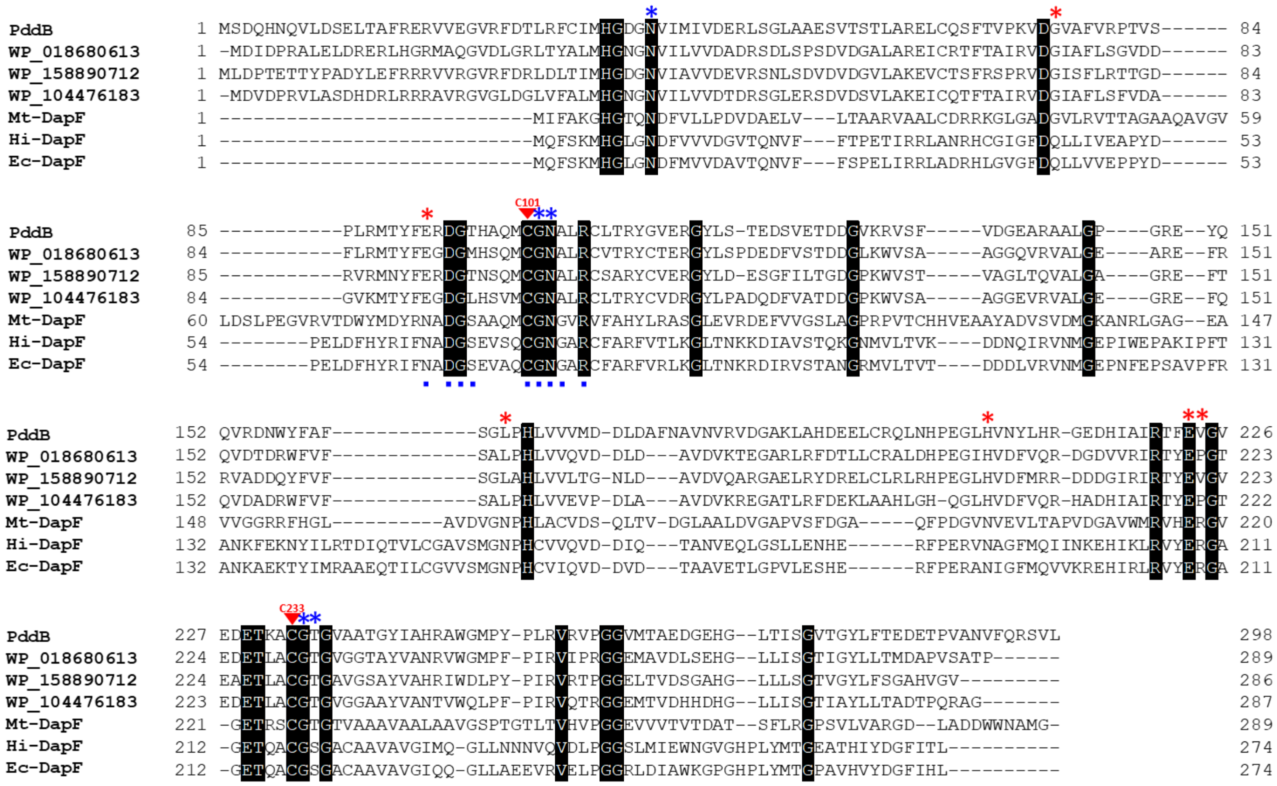

Supplementary Figure S5. Multiple sequence alignment of DabRs and characterized DapFs. Residues corresponding to the DapF active site signature are marked with dots. Residues marked with asterisks in blue are presumably responsible for the recognition of amino- and carboxyl-functionalities at the reactive α-carbon of substrates. Asterisks in red represent key residues potentially responsible for the discriminative recognition of Dab in DabRs. Abbreviations: Hi-DapF, DapF from *Haemophilus influenza* (WP_005655521.1); Ec-DapF, DapF from *Escherichia coli* (WP_001160654.1); all other abbreviations are as defined previously.

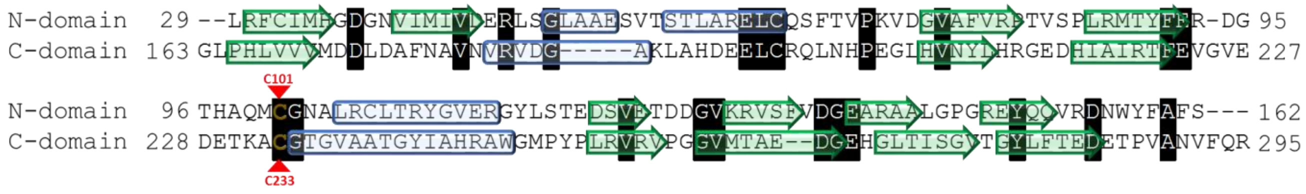

Supplementary Figure S6. Comparative sequence alignment of the PddB domains. The N-terminal domain (position 29 to 162) and the C-terminal domain (position 163 to 295) were aligned. Helices and sheets predicted by using the DSSP algorithm are indicated as blue tubes and green arrows, respectively.
